# An Archaeosome-Adjuvanted Vaccine and Checkpoint Inhibitor Therapy Combination Significantly Enhances Protection from Murine Melanoma

**DOI:** 10.3390/vaccines5040038

**Published:** 2017-10-26

**Authors:** Felicity C. Stark, Risini D. Weeratna, Lise Deschatelets, Komal Gurnani, Renu Dudani, Michael J. McCluskie, Lakshmi Krishnan

**Affiliations:** National Research Council of Canada—Human Health Therapeutics, 1200 Montreal Rd., Ottawa, ON K1A 0R6, Canada; Felicity.Stark@nrc-cnrc.gc.ca (F.C.S.); risini.weeratna@nrc-cnrc.gc.ca (R.D.W.); Lise.Deschatelets@nrc-cnrc.gc.ca (L.D.); Komal.Gurnani@nrc-cnrc.gc.ca (K.G.); Renu.Dudani@nrc-cnrc.gc.ca (R.D.); Michael.McCluskie@nrc-cnrc.gc.ca (M.J.M.)

**Keywords:** checkpoint inhibitor, PD-1, PD-L1, CTLA-4, tumor-infiltrating lymphocyte (TIL), cancer vaccine, CD8^+^ T cell response, archaeosome, prime-boost, B16, liposome, tumor vaccine, effector T cell (TE), effector memory T cell (TEM)

## Abstract

Archaeosomes constitute archaeal lipid vesicle vaccine adjuvants that evoke a strong CD8^+^ T cell response to antigenic cargo. Therapeutic treatment of murine B16-ovalbumin (B16-OVA) melanoma with archaeosome-OVA eliminates small subcutaneous solid tumors; however, they eventually resurge despite an increased frequency of circulating and tumor infiltrating OVA-CD8^+^ T cells. Herein, a number of different approaches were evaluated to improve responses, including dose number, interval, and the combination of vaccine with checkpoint inhibitors. Firstly, we found that tumor protection could not be enhanced by repetitive and/or delayed boosting to maximize the CD8^+^ T cell number and/or phenotype. The in vivo cytotoxicity of vaccine-induced OVA-CD8^+^ T cells was impaired in tumor-bearing mice. Additionally, tumor-infiltrating OVA-CD8^+^ T cells had an increased expression of programmed cell death protein-1 (PD-1) compared to other organ compartments, suggesting impaired function. Combination therapy of tumor-bearing mice with the vaccine archaeosome-OVA, and α-CTLA-4 administered concurrently as well as α-PD-1 and an α-PD-L1 antibody administered starting 9 days after tumor challenge given on a Q3Dx4 schedule (days 9, 12, 15 and 18), significantly enhanced survival. Following multi-combination therapy ~70% of mice had rapid tumor recession, with no detectable tumor mass after >80 days in comparison to a median survival of 17–22 days for untreated or experimental groups receiving single therapies. Overall, archaeosomes offer a powerful platform for delivering cancer antigens when used in combination with checkpoint inhibitor immunotherapies.

## 1. Introduction

Metastatic melanoma is a deadly form of cancer due to its highly invasive nature and limited treatment options. While localized non-melanoma skin cancers can be easily cured by surgical removal with little risk of relapse, unresectable stage IV metastatic melanoma has a 5-year survival rate of less than 5% [1]. Most recently, checkpoint inhibitor immunotherapy to augment T cell responses has been approved as a first-line standard of care treatment for metastatic melanoma; this has drastically improved patient survival. Two recent clinical trials for untreated melanoma using a combination of nivolumab (α-programmed cell death protein-1, α-PD-1) and ipilimumab (α-CTLA-4) reported objective response rates of 53% and 61%, with complete responses seen in 11.5% and 22% of patients [2,3]. To put this in perspective, until recently dacarbazine was the treatment of choice for metastatic melanoma with a 2-year survival rate for patients (with normal LDH levels) of only 40% [4]; however, the inclusion of checkpoint inhibitors nivolumab (α-PD-1) and ipilimumab (α-CTLA-4) has doubled the 1- and 2-year survival rates to 85% and 79%, respectively, [5,6,7] thus marking a sharp turn in cancer therapeutics for melanoma and sparking multiple new clinical trials with checkpoint inhibitor therapy for other cancers [8,9,10,11]. While some cancers are immunogenic enough to sustain anti-tumor CD8^+^ T cell responses in the tumor and periphery, one of the barriers to success with checkpoint inhibitor therapy is thought to be the absence of anti-tumor T cells to non-immunogenic tumors. Since checkpoint inhibitors primarily act on T cells by ”removing the breaks” that prevent their full function, patients without sufficient tumor-specific CD4^+^ and CD8^+^ T cells would be unable to benefit from most checkpoint inhibitor therapies. As a result, there is a renewed interest in cancer vaccines that can stimulate potent CD8^+^ T cell responses. 

Archaeosomes have been shown to activate antigen-specific CD8^+^ T cell responses in multiple pre-clinical studies to date [12]. They exhibit high stability and can not only deliver antigen to antigen presenting cells (APCs) but also possess strong adjuvant properties leading to the induction of potent and long-lasting antigen-specific humoral and cell-mediated immune responses [13,14]. Archaeosomes are liposome vesicles that are composed of glycero-lipids derived from Archaebacteria. Archaeal lipids differ from conventional eubacteria lipids as they possess phytanyl, fully-saturated core lipids, ether linked to the glycerol back-bone. Archaeosomes have traditionally been composed of total polar lipids (TPLs) extracted from archaea and while many different archaebacteria lipids have been tested to formulate archaeosomes, *Methanobrevibacter smithii* was selected for this study as its lipid composition was found to be optimal for eliciting CD8^+^ T cell effector and memory responses when compared to other TPL archaeosomes [13,14]. Archaeosomes can also break tolerance to self-antigens [15], and since they themselves are non-immunogenic they are also highly suitable for homologous prime-boost vaccinations, generating high levels (~45%) of tumor-protective antigen-specific CD8^+^ T cells [16]. However, it has been shown that despite the generation of a large number of tumor-specific effector CD8^+^ T cells, tumor progression can still recur [17,18,19]; this is in part due to tumor-induced immunosuppression that can dampen the cytotoxicity of CD8^+^ T cells [20]. Therefore, many of the current immunotherapeutic strategies aim to not only activate antigen-specific T cells but also to inhibit regulatory receptors with checkpoint inhibitors such as αPD-1, αPD-L1 and/or αCTLA-4. 

In this therapeutic B16-ovalbumin (B16-OVA) solid tumor melanoma mouse model study, ovalbumin was entrapped within archaeosomes composed of TPLs derived from *M. smithii* (MS-OVA archaeosomes) and delivered therapeutically to B16-OVA solid tumor-bearing C57BL/6 mice. The responding CD8^+^ T cell response and phenotype were monitored in the blood and organ compartments. Tumor survival was monitored in a therapeutic MS-OVA archaeosome setting with or without the addition of the checkpoint inhibitors αPD-1, αPD-L1, and/or αCTLA-4. The usefulness of archaeosomes in combination with checkpoint inhibitors to act synergistically providing long-term protection against solid B16-OVA tumors is presented. 

## 2. Materials and Methods

### 2.1. Vaccine Delivery Systems and Route of Immunization

Archaeosomes were prepared from the TPLs of *M. smithii* as described previously [21]. Briefly, the model protein OVA, type VI (Sigma-Aldrich, Oakville, ON, Canada) was encapsulated within archaeosomes by hydrating dried TPLs. Vesicle diameter was reduced to ~100 nm by sonication and assessed with a particle sizer (Nicomp 350, Santa Barbara, CA, USA). Non-entrapped OVA was removed from solution by ultracentrifugation at 327,000× *g*. The supernatant was discarded and the pellet was re-suspended in 1–2 mL of water by gentle vortexing and filtered manually through a 0.45-μm, 25-mm-diameter syringe-driven sterilizing filter. Encapsulated OVA was quantified by SDS-PAGE and the antigen: the lipid ratio was found to be in the range of 20 µg OVA in 0.3–0.7 mg of archaeal lipids. Working stocks for vaccines were diluted in phosphate-buffered saline (PBS; Thermo Fisher Scientific, Ottawa, ON, Canada) (20 µg OVA/100 µL PBS). Mice received a subcutaneous (s.c.) injection of MS-OVA (20 µg OVA entrapped in archaeosomes) at the base of the tail at various time-points after tumor challenge (see Section 2.3 below). Numbers of animals per group and vaccination regimen are indicated in the figure legends. 

### 2.2. Mouse Strains and Adoptive Cell Transfer

Here, 6–8 week old female C57BL/6 and OT.1 TCR transgenic mice (with CD8^+^ cells expressing the OVA_257–264_ TCR) were obtained from Jackson Laboratory (Bar Harbor, ME, USA). Mice were maintained at the small animal facility of the National Research Council Canada (NRC) in accordance with the guidelines of the Canadian Council on Animal Care. All animal use protocols were approved by the NRC Animal Care Committee (Protocol 2011.24). For results shown in Figures 4 and 5, mice received an adoptive transfer of 10^5^ OT.1 splenocytes 2 days after B16-OVA tumor injection to increase the frequency of circulating OVA-CD8^+^ T cells as previously described [22].

### 2.3. Tumor Model (B16-OVA, Melanoma)

B16F0-OVA (expressing plasmid-derived full-length OVA) cells were obtained from Dr. Edith Lord (University of Rochester, Rochester, New York) and cultured as described previously [23,24]. Solid tumors were induced with s.c. injection of 1 × 10^6^ B16-OVA cells. From day 5 onwards, a detectable solid tumor was measured using Digimatic Digital calipers (Mitutoyo 500-196, Aurora, IL, USA). Tumor size, expressed in mm^2^, was obtained by multiplication of diametrically perpendicular measurements. Animals were monitored for long-term survival. However, in order to minimize pain and discomfort, mice were euthanized when tumors reached 300 mm^2^.

### 2.4. Assessment of In Vivo Cytolytic Activity

In vivo cytolytic activity of CD8^+^ T cells was enumerated as described previously [25]. Briefly, a mixture of SIINFEKL peptide-pulsed and non-peptide-pulsed CFSE (2 µM) labeled naive splenocytes was injected at a 1:1 ratio at different time points after MS-OVA administration in both tumor bearing or non-tumor bearing mice. At 24 h post cell injection, spleens were removed from recipient mice and analyzed by flow cytometry for loss of target cells relative to non-target control cells, and the percentage of in vivo killing was calculated. 

### 2.5. Detection of OVA- Specific CD8^+^ T Cells

At various time points (pre-vaccination, 1-week post-vaccination, and at regular intervals throughout the course of the study) blood (50–100 µL) was collected via submandibular venipuncture for FACS analysis. At 33 and 37 days post tumor injection in MS-OVA treated mice blood, tumor, spleen, and lymph nodes were collected and processed for FACS analysis. Spleen and blood samples were processed as described previously [16]. Lymph nodes were processed by mashing between the frosted ends of two glass slides in RPMI 1640 medium (Invitrogen, Life Technologies, Grand Island, New York, NY, USA) supplemented with 8% fetal bovine serum (FBS) (HyClone Laboratories, Logan, UT, USA) (R8) and passed twice through a 45-μm Falcon cell strainer. Cells were washed by centrifugation at 400× *g* for 8 min and finally re-suspended in 0.5 mL of R8 medium. Subcutaneous tumors were excised in R8 medium, cut into small pieces, and digested with sterile dissociation cocktail comprising a final concentration of 1 mg/mL of collagenase type 4 (Worthington Biochemical Corporation, Lakewood, NJ, USA), and 0.1 mg/mL of hyaluronidase (Sigma-Aldrich, Oakville, ON, Canada). Samples were incubated for 1 h in a 37 °C shaking incubator, and then passed through a 45-μm falcon cell strainer (BD Biosciences, Franklin Lakes, NJ, USA). Cells were centrifuged at 400 × *g* for 8 min at RT and resuspended in 5 mL PBS + 1% BSA. Cells were strained and washed repeatedly until there were no visible clumps. Lymphocytes were isolated by Percoll^TM^ density gradient centrifugation. Briefly, a gradient was prepared by successively layering 40% and 70% Percoll density solutions, and cells in PBS were layered on top. Samples were centrifuged at 800× *g* for 25 min at 4 °C. The lymphocytes were collected from the lower interphase, thoroughly washed in PBS, centrifuged, and resuspended in R8 medium. The low-density tumor cells were found concentrated in the upper interphase. Live cell number was enumerated by trypan blue exclusion using a hemocytometer. Single cell suspensions were first blocked with an anti-Fc receptor antibody (α-CD16) for 5 min at 4 °C. Whole blood and single cell suspensions were stained with antibodies against CD8^+^ , IL-7Rα, CD62L, and PD-1 as well as with the MHC tetramer H-2K^b^OVA. All antibodies were obtained from BD Biosciences (Mississauga, ON, Canada). The H-2K^b^OVA-tetramer was obtained from Beckman Coulter (Mississauga, ON, Canada). Blood samples were treated with RBC lysing buffer (Sigma-Aldrich Oakville, ON, Canada) after antibody staining. Cells were washed with PBS, fixed with 0.5% paraformaldehyde, and acquired on a BD FACS Canto analyzer (Becton, Dickinson and Company, Franklin Lakes, NJ, USA). FSc vs. SSc gating was used to locate lymphocytes, exclude doublets, and exclude debris and dead cells. A further gate was set to identify CD8^+^, and the MHC tetramer H-2K^b^OVA^+^ cells. Single stained antibody controls were used to set compensation values between channels to prevent overlapping signals creating false-positives. Additionally, fluorescence minus one (FMO) gating controls were used. Flow cytometry data were analyzed using the FACS Diva^®^ software (Becton, Dickinson and Company, Franklin Lakes, NJ, USA). 

### 2.6. Checkpoint Inhibitor Combination Therapy

C57BL/6 mice were given 5 × 10^5^ B16-OVA cells s.c. in the dorsal flank; 3, 8, and 18 days later 20 μg MS-OVA was injected s.c. at the base of the tail away from the tumor site. A total of 100 μg α-CTLA-4, Clone 9D9 (BioXcell, West Lebanon, NH, USA) was given s.c. alongside the archaeosome treatment (days 3, 8, and 18). A total of 250 μg each of α-PD-1, RMP1-14 (BioXcell, West Lebanon, NH, USA) and α-PD-L1, 10F.9G2 (BioXcell, West Lebanon, NH, USA) was given i.p. on days 9, 12, 15, and 18. Rationale for timing and injection site choice for checkpoint inhibitors: α-CTLA-4 was given alongside the archaeosome vaccine s.c. so that it would drain to the same lymph nodes and act on the same CD8^+^ T cells that were being activated by the vaccine. α-PD-1 and α-PD-L1 were given in parallel i.p. at the time when tumors were thought to be growing so they could systemically act on augmenting CD8^+^ T cells responses to the tumor. 

## 3. Results

### 3.1. MS-OVA Therapy Against B16-OVA Melanoma Induces OVA-CD8^+^ T Cells and Modest Tumor Protection

**Figure 1:**
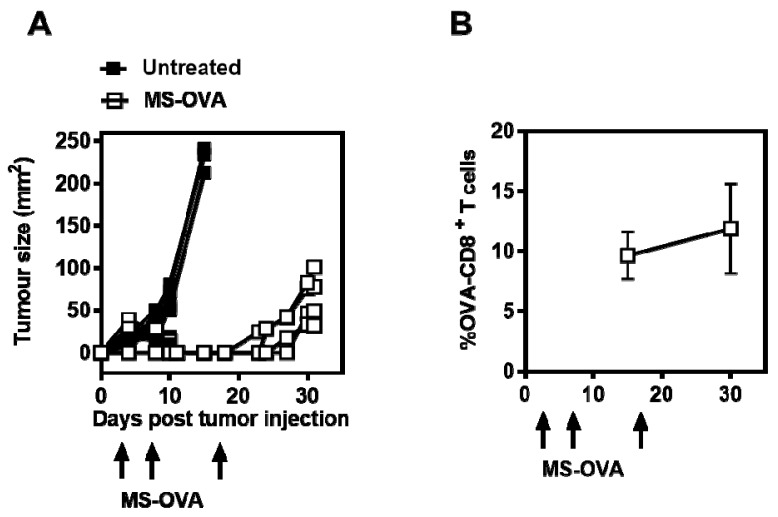
Therapeutic treatment of subcutaneous B16-ovalbumin (B-16-OVA) melanoma with *Methanobrevibacter smithii*-OVA (MS-OVA). C57BL/6 mice were given 10^6^ B16-OVA melanoma tumor cells subcutaneously (s.c.) in the dorsal flank. On days 4, 8, and 18 after tumor injection, 20 μg MS-OVA was injected s.c. at the base of the tail away from the tumor site. (**A**): Tumor growth in individual mice is shown for vaccinated mice and non-vaccinated mice; (**B**): The mean frequency ± SD of OVA-CD8^+^ T cells is shown at two time points for vaccinated mice (*n* = 4/group). This was repeated twice.

The infiltration of a large number of tumor antigen-specific CD8^+^ T cells into tumor sites has been correlated with an improved outcome in colorectal, epithelial, ovarian, and metastatic tumors [26,27,28]. As archaeosomes have previously been shown to induce antigen-specific CD8^+^ T cell responses, we first evaluated the efficacy of MS-OVA in a therapeutic tumor model. C57BL/6 mice received 10^6^ B16-OVA s.c in the dorsal flank, followed by MS-OVA s.c. at the base of the tail on days 4, 8, and 18. Non-vaccinated control mice succumbed to tumor burden within 10–15 days, whereas therapeutic MS-OVA treatment extended the median survival to 31 days (Figure 1A). MS-OVA given in a tumor-bearing mouse was capable of activating and expanding OVA-CD8^+^ T cells from 0.1% (data not shown) up to 10% of all circulating CD8^+^ T cells in the blood sampled on days 15 and 30 after tumor challenge (Figure 1B). However, despite the presence of a large number of OVA_257–264_-specific CD8^+^ T cells, tumor growth continued. 

**Figure 2:**
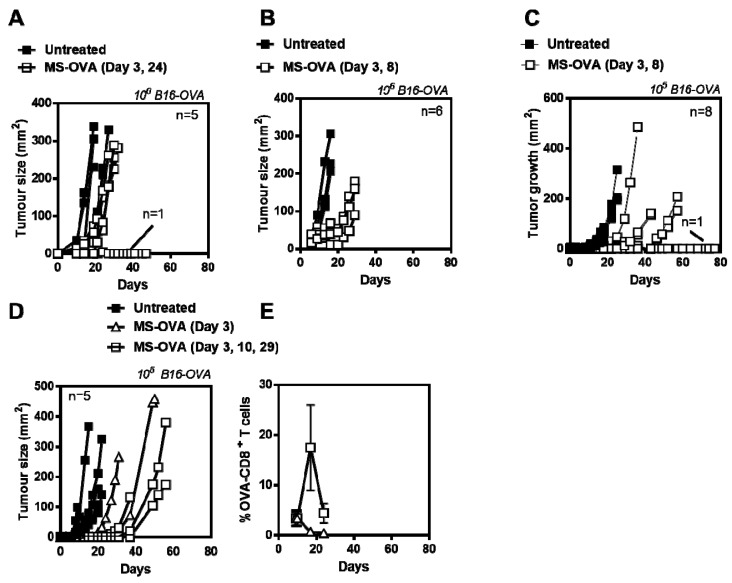
Optimizing MS-OVA treatment. C57BL/6 mice were given 10^6^ B16-OVA tumor cells s.c. in the dorsal flank, MS-OVA was given s.c. at the base of the tail on (**A**), day 3 and 24 or (**B**) day 3 and 8, and tumor growth was measured over time. Alternatively, a reduced number of tumor cells, 10^5^ of B16-OVA, were injected followed by MS-OVA treatment on (**C**) day 3 and 8, (**D**) day 3, or day 3, 10 and 29, and tumor growth was measured. The mean frequency ± SD of OVA-CD8^+^ T cells was compared in mice treated with MS-OVA on day 3 vs. days 3, 10, and 29 (**E**).

In an attempt to improve responses, we evaluated different immunization schedules (Day 3 and 24; Day 3 and 8), however, while all vaccinated mice had smaller tumors than control mice, all but one succumbed to tumor burden and no discernible difference in protection was observed (Figure 2A,B). Since tumors grew so aggressively with 10^6^ B16-OVA tumor cells (median survival time of 10–15 days with a small therapeutic window) we also evaluated using lower tumor cell numbers. A starting dose of 10^4^ B16-OVA tumor cells resulted in slower tumor growth but not all animals developed tumors (data not shown). Likewise, with 10^5^ B16-OVA cells, the tumors also took longer to develop, however the variability between vaccinated mice also increased (Figure 2C). We also compared mice vaccinated on a single (day 3) or triple occasion (day 3, 10, 29) and while OVA-CD8^+^ T cell frequency increased with multiple vaccinations (Figure 2E) all mice had similar survival outcomes Figure 2D). Thus, while MS-OVA treatment increased time to tumor progression compared to control animals, there was no overall survival benefit with increased number of immunizations despite there being an increased frequency of OVA_257–264_-specific CD8^+^ T cells in the blood. This suggested that quality of CD8^+^ T cells (i.e., cytolytic capability) may have been impaired. 

### 3.2. OVA-CD8^+^ T Cell Cytotoxicity Is Impaired in Mice Bearing Solid B16-OVA Tumors

To investigate whether functionality of OVA-CD8^+^ T cells induced by MS-OVA was impaired in B16-OVA tumor bearing mice, we used an in vivo cytotoxicity assay whereby vaccinated mice were injected with CFSE-stained target cells previously pulsed with OVA-peptide and non-pulsed cells as a control and the percentage of in vivo killing was calculated 24 h later based on the survival proportions of target cell populations. 

**Figure 3:**
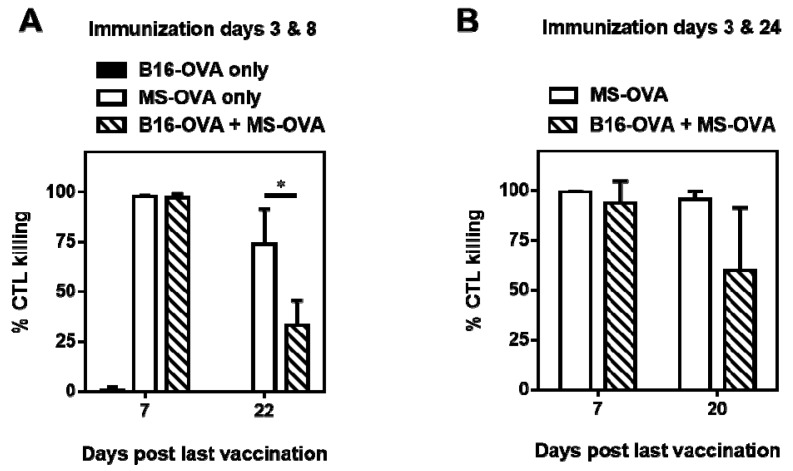
In vivo cytotoxicity assay during immunotherapy with MS-OVA. Naïve C57BL/6 mice were given 10^6^ B16-OVA tumor cells s.c. on day 0 in the dorsal flank. A total of 20 μg MS-OVA was administered s.c. at the base of the tail away from the tumor site. At 1 week or 3 weeks post boost, CFSE-stained target splenocytes were injected intravenously (i.v.); 24 h later recipient spleens were removed and processed to measure in vivo cytolytic killing. The different vaccination timings on day 3 and 8 (**A**), or 3 and 24 (**B**) were carried out in two separate experiments. The graphs show in vivo cytolytic killing for individual animals and the group mean ± SD (*n* = 3–5/timepoint/group) at 1 week and 3 weeks post boost. Mean ± SD, *n* = 3–5/timepoint/group. * *p* = 0.0308 Unpaired two-tailed *t* test was performed with GraphPad Prism software.

Following administration of MS-OVA on an accelerated dosing schedule (day 3 and 8), at one week post the second dose, 94 to 98% of CFSE stained target cells were killed relative to non-target cells in both tumor and non-tumor-bearing mice (Figure 3A). As expected, tumor-bearing naïve non-immunized mice did not exhibit any killing of target cells (Figure 3A) indicating that the CD8^+^ T cell response to the endogenous tumor immunogen was negligible. From day 7 to 22, a loss of cytolytic ability was observed in both non-tumor bearing and tumor-bearing animals that were administered MS-OVA (Figure 3A). Since previous studies have shown that a high level (>80%) of target-specific cytolytic ability can be maintained for extended periods of time (~100 days post vaccination) with a more extended immunization schedule (day 3 and 24) [29], it is possible that the boosting schedule chosen (day 3 and 8) may not have been efficient to induce sustained cytolytic activity. Therefore, in a separate experiment, we also evaluated a more extended immunization schedule (day 3 and 24). Using this schedule, non-tumor bearing vaccinated mice maintained antigen-specific cytolytic ability by CD8^+^ T cells for the duration of the study as previously observed [29], however a decrease in cytolytic function was still observed in tumor-bearing mice over time (Figure 3B), suggesting impaired CD8^+^ T cell function by tumor suppressive mechanisms. It is also possible that the immunosuppression observed may not be antigen-dependent as we did not test this in a B16 model (without an OVA antigen). Since the modulation of timing of therapy on its own could not improve CD8^+^ T cell function, further investigation into the phenotype of responding OVA-CD8^+^ T cells was warranted. 

### 3.3. Phenotype and Frequency of Responding OVA-CD8^+^ T Cells

We have previously demonstrated that the numbers of endogenous OVA-CD8^+^ T cells induced by MS-OVA can be low, making it difficult to track the phenotype of subpopulations over time. Therefore, the pool of available OVA-CD8^+^ T cells was increased by adoptively transferring splenocytes from an OT.1 mouse in which CD8^+^ T cells are specific for the OVA epitope SIINFEKL [30,31]. Since B16-OVA is considered an immunogenic tumor, we first investigated whether the tumor-derived OVA antigen could activate adoptively transferred OT.1 CD8^+^ T cells. Mice were injected with 10^6^ B16-OVA cells, and 3 days later 10^4^, 10^5^, or 10^6^ OT.1 splenocytes were injected intravenously and the frequency of OVA-CD8^+^ T cells was monitored over time (Appendix Figure A1A). The overall frequency of OVA_257–264_-specific CD8^+^ T cells was low (0.1–0.6%) in all tested doses, and no statistically significant increase occurred over time. Importantly, there were no differences in B16-OVA tumor growth patterns in mice adoptively transferred with OT.1 cells (Appendix Figure A1B).

**Figure 4:**
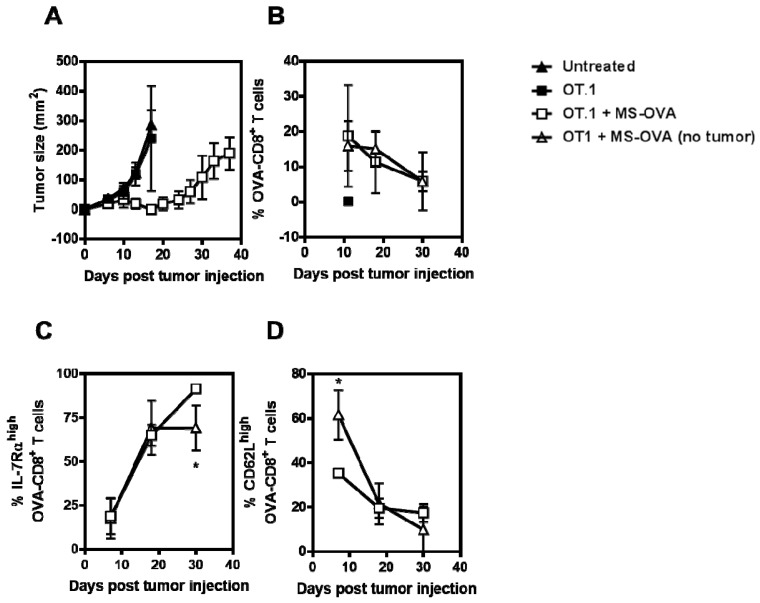
Frequency and phenotype of responding OVA_257-264_-specific CD8^+^ T cells. C57BL/6 mice were given 10^6^ B16-OVA tumor cells s.c. in the dorsal flank. Two days later, 10^5^ OT.1 splenocytes were given intravenously. On days 3 and 8 after tumor injection, 20 μg MS-OVA was injected s.c. at the base of the tail away from the tumor site. (**A**) Tumor growth was measured over time. At varying time points blood was collected for FACS analysis of (**B**) OVA-CD8^+^ T cells, and their expression of (**C**) CD62L and (**D**) IL-7Rα. Mean ± SD, *n* = 5. Unpaired two-tailed *t* test with Welch’s correction for unequal variance (**C**) * *p* < 0.05 (**D**) * *p* < 0.05.

To assess the phenotype of responding OVA-CD8^+^ T cells in this MS-OVA therapy model, C57BL/6 mice were first injected with 10^6^ B16-OVA cells, followed by the adoptive transfer of 10^5^ OT.1 splenocytes (~10 to 20% are OVA-CD8^+^ T cells, data not shown) 2 days later. Mice were then therapeutically treated with 20 µg MS-OVA on day 3 and 8 and blood samples collected at various time-points (days 11, 18, and 30). As previously seen, immunization with MS-OVA decreased tumor growth and increased survival (median survival of 37 days) compared to non-vaccinated controls (median survival of 13 days) (Figure 4A). The presence of tumor did not appear to alter the frequency of responding OVA_257–264_-specific CD8^+^ T cells in the blood compared to a non-tumor-bearing vaccinated mouse (Figure 4B). CD62L and IL-7Rα expression was also monitored with respect to responding OVA-CD8^+^ T cells to assess the quality of the response as both CD62L and IL-7Rα are rapidly downregulated in effector CD8^+^ T cells and remain downregulated as long as cells are exposed to their cognate antigen. Re-upregulation of IL-7Rα indicates a progression to memory phenotype. Expectedly, CD62L expression on CD8^+^ T cells in the blood of tumor-bearing mice was lower compared to non-tumor bearing mice; yet at later time-points, a similar level of expression of this marker was observed in both tumor and non-tumor bearing mice (Figure 4C). IL-7Rα expression in OVA-CD8^+^ T cells was statistically higher in tumor-bearing mice by day 30 (Figure 4D), but may be a result of IL-7Rα^low^ CD8^+^ T cells trafficking to the tumor site to exert effector functions. 

### 3.4. Tumor-Infiltrating CD8^+^ T Cells Express PD-1 Following Therapeutic MS-OVA Treatment

**Figure 5:**
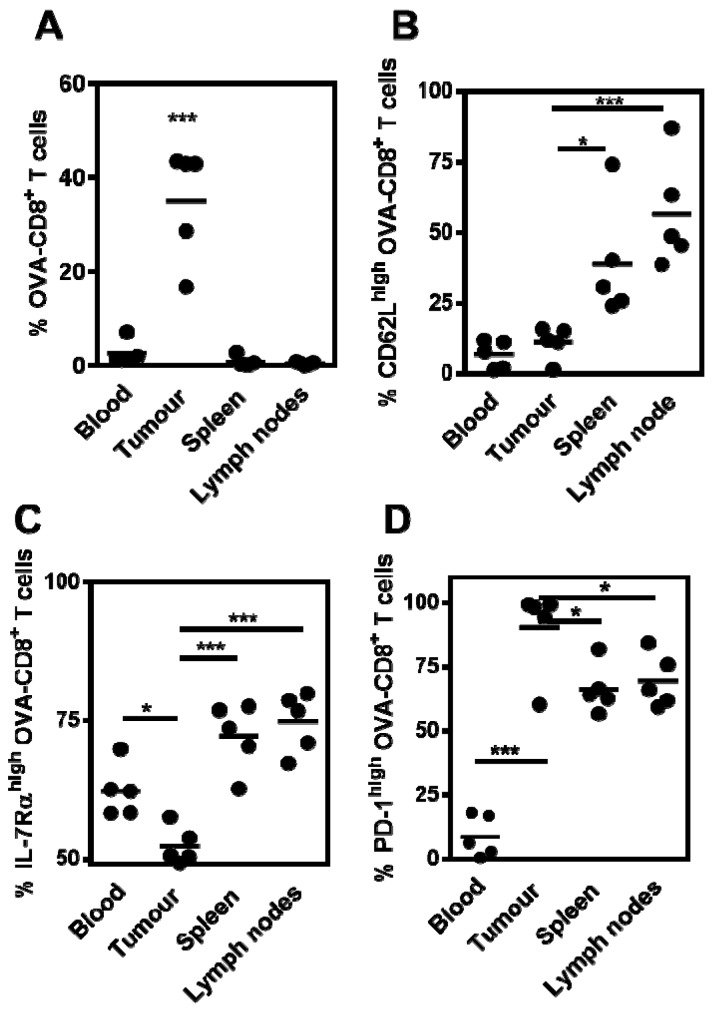
Phenotype and location of responding OVA_257–264_-specific CD8^+^ T cells in MS-OVA-vaccinated tumor bearing mice. C57BL/6 mice were given 10^6^ B16-OVA tumor cells s.c. in the dorsal flank. Two days later, 10^5^ OT.1 splenocytes were given intravenously. On days 3 and 8 after tumor injection, 20 μg MS-OVA was injected s.c. at the base of the tail away from the tumor site. At days 33 and 37 post tumor injection, blood was collected and organs excised and processed to single cell suspensions for FACS analysis. The % of OVA-CD8^+^ T cells (**A**) and the frequency of CD62L^high^ (**B**), IL-7Rα^high^ (**C**) or PD-1^high^ (**D**) was measured (mean ± SD, *n* = 5). One-way ANOVA and Dunnet’s post tests are as follows: *** (*F*(3,16) = 37.69, *p* < 0.0001), tumor vs. all *** *p* < 0.0001), *** (*F*(3,16) = 13.05, *p* < 0.0001), tumor vs. spleen * *p* < 0.05, tumor vs. lymph nodes *** *p* < 0.0001. *** (*F*(3,16) = 21.08, *p* < 0.0001), tumor vs. blood * *p* < 0.05, tumor vs. spleen *** *p* < 0.0001, tumor vs. lymph nodes *** *p* < 0.0001. *** (*F*(3,16) = 44.43, *p* < 0.0001), tumor vs. blood *** *p* < 0.0001, tumor vs. spleen * *p* < 0.05, tumor vs. lymph nodes * *p* < 0.05.

Programmed cell death protein-1 (PD-1) is upregulated on activated CD8^+^ T cells [32] and its maintained expression mediates the down-regulation of CD8^+^ T cell cytotoxicity and is thought to play a role in the immune evasion of tumors [33]. To characterize the phenotype of OVA-CD8^+^ T cells after MS-OVA therapy, mice were pre-dosed with OT.1 splenocytes to increase the basal frequency of OVA-specific CD8^+^ T cells, thereby increasing the frequency of responding OVA-specific CD8^+^ T cells. After tumor development, mice were sacrificed and blood, spleen, tumor and lymph nodes were taken to analyze tissue-resident OVA-specific CD8^+^ T cells and their phenotype. In MS-OVA treated mice, there was an increased frequency of OVA-specific CD8^+^ T cells in the tumor compared to the spleen, tumor-draining lymph nodes and the blood (Figure 5A). Surface expression of CD62L and IL-7Rα was significantly higher in OVA-specific CD8^+^ T cells in the spleen and lymph nodes compared to the tumor (Figure 5B,C). Most, interestingly a significantly increased expression of PD-1 was observed in infiltrating OVA-specific CD8^+^ T cells compared to other sites (Figure 5D). Additionally, PD-1 expression was significantly lower on blood resident CD8^+^ T cells. Since PD-1 upregulation has been correlated with diminished T cell function [34], this could represent a loss of tumor control and may account for loss of CTL function and resurgence of tumor after MS-OVA therapy. 

### 3.5. Combination Checkpoint Inhibitor Therapy with MS-OVA

**Figure 6:**
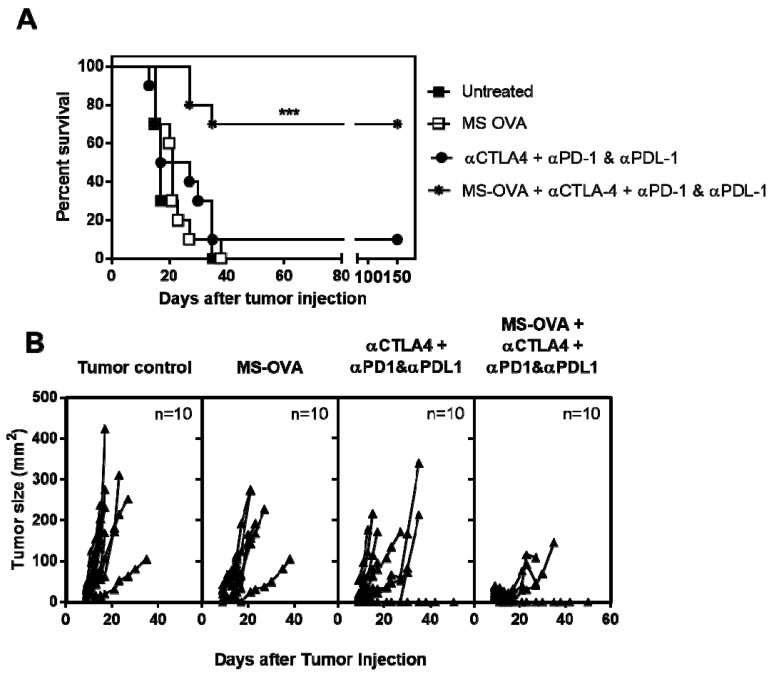
MS-OVA therapy in combination with checkpoint inhibitor therapy significantly improves survival outcome. C57BL/6 mice were given 5 × 10^5^ B16-OVA tumor cells s.c. in the dorsal flank; 3, 8, and 18 days later 20 μg MS-OVA was injected s.c. at the base of the tail away from the tumor site. In total 100 μg αCTLA-4 (Clone 9D9) was given s.c. alongside the archaeosome vaccination. Then, 250 μg each of αPD-1 (clone: RMP1-14) and αPD-L1 (clone: 10F.9G2) was given i.p on days 9, 12, 15, and 18. Log-rank Mantel Cox test, *** *p* < 0.001. This study was repeated to confirm results.

To address the question of whether T cell inhibitory receptors or ligands, such as PD-1, CTLA-4 and PD-L1, were responsible for the impaired OVA-CD8^+^ T cell function and resumed tumor growth, we next evaluated whether antibody therapy targeting these molecules would enhance the efficacy of MS-OVA therapy in B16-OVA tumor-bearing mice. B16-OVA bearing C57BL/6 mice were treated on day 3, 8, and 18 after tumor injection with both MS-OVA (day 3, 8 and 18) and/or αCTLA-4 (day 3, 8, and 18) and/or αPD-1/αPD-L1 (day 9, 12, 15, and 18). We chose to use both αPD-1 and αPD-L1 to block the entire PD-1/PD-L1 pathway as each molecule is known to bind to other ligands mediating immunosuppression [35,36]. Survival was significantly improved when MS-OVA archaeosomes were used in combination with checkpoint inhibitors with best responses obtained with a combination of MS-OVA archaeosomes + αCTLA-4/αPD-1/αPD-L1 (Figure 6). Indeed, using this combination, 70% of animals survived beyond 80 days compared to a median survival of 21 and 22 days for MS-OVA or αCTLA-4/αPD-1/αPD-L1 checkpoint inhibitor therapy alone, respectively. Weaker responses were obtained with MS-OVA + αCTLA-4 (median survival = 32 days), MS-OVA + αPD-1/αPD-L1 (median survival = 34 days ), αCTLA-4 alone (median survival = 23 days), or αPD-1/αPD-L1 (median survival = 18.5 days) (data not shown). 

## 4. Discussion

The presence of anti-tumor CD8^+^ T cells within a tumor can be a positive prognostic factor which correlates with the presence of intratumoral granzyme B, IFN-γ and IL-2, hallmarks of cytolytic CD8^+^ T cell responses [28,37,38]. However, while large numbers of CD8^+^ T cells can be found in some types of tumors, tumor progression often still persists; in these cases inhibitory mechanisms such as the immunosuppressive CD8^+^ T cell receptors, PD-1, and CTLA-4 as well as tumor-expressed PD-L1 are often involved. In investigating the usefulness of MS-OVA to induce potent CD8^+^ T cell responses in a therapeutic tumor model, we observed that while MS-OVA could induce antigen-specific CD8^+^ T cells in tumor-bearing mice which resulted in a decrease in tumor burden and even a short tumor-free period, tumor recurrence occurred and mice eventually succumbed to tumor burden. To determine whether responses could be improved by increasing the frequency of anti-tumor CD8^+^ T cells, we evaluated different immunization schedules and showed that while boosting with MS-OVA could increase the frequency of CD8^+^ T cells, overall tumor protection in the therapeutic setting did not significantly improve compared to a single dose vaccine. For example, a similar rate of tumor occurrence, was obtained with 1, 2, or 3 immunizations regardless of boosting interval or level of OVA_257–264_ specific CD8^+^ T cell frequency. Since it was possible that tumor challenge dose was simply too high for any anti-tumor immune responses to be effective, we reduced the tumor challenge dose from 10^6^ to 10^5^ B16-OVA cells. However, while the onset of tumor was expectedly delayed, no difference in vaccine efficacy could be achieved. Likewise, it was possible that the time interval between priming and boosting with MS-OVA was too short to propagate large numbers of OVA_257–264_-specific CD8^+^ T cells and so we increased this in an attempt to generate higher frequencies of CD8^+^ T cells. Indeed, an in vivo cytotolytic assay confirmed that in the absence of tumor, a second dose of MS-OVA at an early time-point (5 days after priming) reduced the maintenance of CD8^+^ T cell cytotoxicity, whereas delaying the second dose to a later time-point (21 days later) maintained cytotoxicity at ~100% even up to 3 weeks after the booster dose. However, when this was evaluated in tumor-bearing mice, there was a decrease in in vivo cytolytic activity regardless of time interval prior to boosting and no enhancement of tumor protection. It is possible that delaying a booster vaccination in a tumor-bearing mouse by 2 weeks failed because tumor burden was much too large by this time-point and that CD8^+^ T cell cytotoxicity was impaired by tumor-suppressive mechanisms, highlighting the requirement not only for robust anti-tumor immune responses but also combinatorial approaches to mitigate tumor-mediated immunosuppression. It is possible that responses may have been improved by using a heterologous prime-boost regime as has been reported by other groups [39,40] however, we did not evaluate this as we have previously shown that archaeosomes work effectively for both priming and boosting and are capable of inducing high levels of antigen-specific CD8^+^ T cells [15,16,29]]. Moreover this approach is not likely to overcome immunosuppression in the tumor. Immune suppressive mechanisms can be evoked by tumors that can reduce the effectiveness of anti-tumor CD8^+^ T cells. These mechanisms include the up-regulation of inhibitory receptors such as PD-1 on CD8^+^ T cells [41,42]. In our study, tumor-bearing mice treated therapeutically with MS-OVA generated OVA-specific CD8^+^ T cell responses in the blood that when compared to non-tumor bearing mice had a slightly lower number of IL-7Rα high cells and the same low number of CD62L^high^ cells at a time-point when the tumors were measured to be growing. This phenotype indicates a mixed population of effector CD8^+^ T cells (T_E_) and effector memory CD8^+^ T cells (T_EM_). In a prophylactic vaccination setting a T_E_/T_EM_ phenotype is thought to be less effective compared to a predominant central memory CD8^+^ T cell (T_CM_) phenotype due to their limited proliferation potential [43], however in a therapeutic setting with the continued persistence of OVA antigen in the tumor it is not unexpected that they express an activated effector phenotype and others have shown this to correlate with a higher level of PD-1 expression [44]. 

In MS-OVA treated tumor bearing mice, tumor growth was suppressed for a short period of time, however when tumor returned, CD8^+^ T cells located within the tumor tissue, spleen, and lymph nodes notably exhibited high levels of PD-1 expression (90%, 66%, and 70%, respectively). The induction of inhibitory receptors after vaccination has previously been shown in both preclinical models and in humans and so was not entirely unexpected [45,46]. These increases of PD-1 expression in CD8^+^ T cells highlight a possible cause for the relapse of tumor growth in this vaccination scenario, suggesting the use of a combination therapy that includes targeting PD-1 with antibody therapy. The use of a blocking antibodies against PD-1 can rescue CD8^+^ T cell lytic abilities [47]. Similarly, targeting PD-1, PD-L1, and CTLA-4 with monoclonal antibodies has been shown to reverse CD8^+^ T cell exhaustion and enhance lytic capabilities [48,49,50] as well as promote tumor shrinkage and survival for many cancer patients [51,52,53]. Successes in clinical trials have led to the approval of a number of checkpoint inhibitor therapies by national health authorities (US Food and Drug Administration, Health Canada etc.) for use against multiple tumor indications [7,53]. Checkpoint inhibitor therapy has even been approved for use as a monotherapy, its mode of action being to ‘remove the brakes’ on a patient’s existing tumor-specific CD8^+^ T cells. However, these therapies, which include αPD-1, αPD-L1, and αCTLA-4, have been observed to be ineffective in certain circumstances that include immunologically cold tumors where physical barriers impede immune cell invasion, or when tumor-specific T cells are not present at all. In the latter situation, a good approach would be to combine checkpoint inhibitor therapy with a vaccine that can boost the level of circulating tumor-specific T cells. Indeed, a synergy between antibodies blocking inhibitory pathways and various types of cancer vaccines including peptide vaccines, live vectors, dendritic cell targeting inert vectors, cellular vaccines, and DNA vaccines has previously been reported [54]. Since we observed that following vaccination there was an increased expression of PD-1 in tumor infiltrating CD8^+^ T cells, we sought to evaluate whether the efficacy of MS-OVA could be improved by combining with commercially available antibodies against PD-1 and other inhibitory receptors. Treatment of B16-OVA with all three checkpoint inhibitors alone (i.e., without vaccines) did not enhance survival greater than for the control. This was not unexpected since B16-OVA does not induce tumor-specific T cells, and for antibodies which block inhibitory pathways to be effective, there is a requirement for pre-existing anti-tumor CD8^+^ T cells at the tumor site [55]. When MS-OVA was combined with αCTLA-4 or with a combination of both αPD-1 and αPD-L1, there was a slight improvement in survival although over 90% of animals did eventually succumb to tumor (data not shown). However, when a combination of MS-OVA with αCTLA-4, αPD-1, and αPD-L2 was used, 70% of animals survived beyond 100 days compared to a median survival of 21 and 22 days for MS-OVA or αCTLA-4/αPD-1/αPD-L1 checkpoint inhibitor therapy alone, respectively. While this is the first report of checkpoint inhibitors in combination with an archaeosome-based vaccine, our findings also support previous studies using the B16-OVA model in mice whereby a *Salmonella* *typhimurium*-based vaccine was used to deliver the OVA SIINFEKL epitope and resulted in 32% rejection of long-established tumors which could be increased to 80% when the vaccine was combined with αPD-L1 or αPD-L1 plus αCTLA-4 [56]. Likewise, an Fms-like tyrosine kinase 3 (Flt3)-based tumor cell vaccine combined with blocking antibodies directed against CTLA-4, PD-1, and PD-L1 resulted in rejection of B16 melanoma tumors in 65% of mice, compared to 10% with vaccine plus αCTLA-4 alone, 25% with vaccine plus αPD-1 alone and 50% with vaccine plus αCTLA-4 combined with αPD-1 [57]. Dual blockade of both PD-1 and CTLA-4 in combination with tumor vaccine has also been shown to enhance tumor rejection in other mouse tumor models such as CT26 colon carcinoma and ID8-VEGF ovarian carcinoma [58]. A number of other checkpoint inhibitor-targeting antibodies have also been previously used with cancer vaccines, including α-Tim-3, α-Tim-4, α-OX40 [54] and it is likely that these would also be beneficial if used in combination with our archaeosome-based vaccine. Indeed future studies with archaeosomes in tumor therapy will involve testing of novel checkpoint inhibitors as well as testing of these combinations with more clinically relevant tumor associated antigens such as Trp2, for which archaeosomes have previously been shown to break tolerance to [15]. Archaeosomes possess extremely high stability due to their unique chemical constitutions and have been shown to have a high safety profile and to activate antigen-specific CD8^+^ T cell responses in multiple studies to date, making them an appropriate choice for adjuvanting an anti-tumor vaccine [12]. Herein, the combination of an archaeosome-based vaccine and checkpoint inhibitors directed against PD-1, PD-L1, and CTLA-4 had a clear synergistic effect, improving overall survival of B16-OVA-bearing mice being treated therapeutically, and shows a clear benefit for the use of therapeutic vaccines such as archaeosomes in modern cancer therapy regimes. 

## 5. Conclusions

Herein, we have shown that an archaeosome-based vaccine alone will induce OVA-CD8^+^ T cells capable of providing short-term protection in therapy against solid subcutaneous B16-OVA in mice and that this correlated with a high level of PD-1 expression on tumor-infiltrating OVA-CD8^+^ T cells. However, when this was combined with antibodies directed against the CD8^+^ T cell inhibitory receptors PD-1 and CTLA-4 as well as the inhibitory ligand PD-L1 we were able to obtain long-lasting protection from the B16-OVA tumor in 70% of C57BL/6 mice. Thus, an archaeosome-based vaccine in combination with checkpoint inhibitor therapy offers an attractive approach to promote long-term tumor protection. 

## Figures and Tables

**Figure A1:**
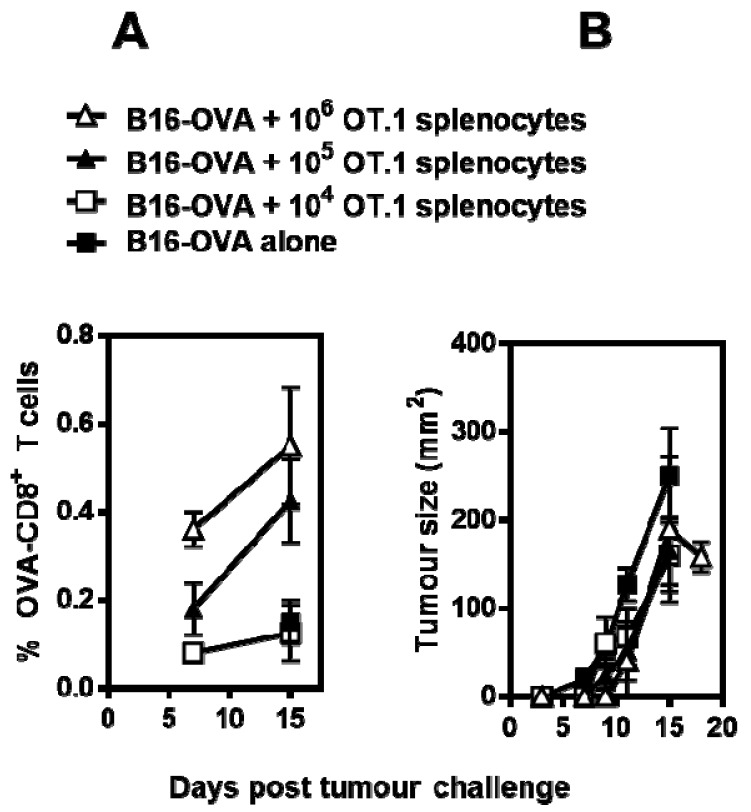
Basal level of circulating OVA-CD8^+^ T cells and the optimization of adoptively transferred OT.1 splenocytes for tracking OVA-CD8^+^ T cell responses. C57BL/6 mice were challenged s.c. with 10^6^ B16-OVA. 3 days later PBS control, 10^4^, 10^5^ or 10^6^ OT.1 splenocytes are injected i.v. (**A**) Blood samples are taken to monitor OVA_257–264_ specific CD8^+^ T cells; (**B**) Tumor size was monitored over time. (Mean ± SE, n = 5/group).
